# Personalized 5-Year Prostate Cancer Risk Prediction Model in Korea Based on Nationwide Representative Data

**DOI:** 10.3390/jpm12010002

**Published:** 2021-12-21

**Authors:** Yohwan Yeo, Dong Wook Shin, Jungkwon Lee, Kyungdo Han, Sang Hyun Park, Keun Hye Jeon, Jungeun Shin, Aesun Shin, Jinsung Park

**Affiliations:** 1 Department of Family Medicine and Supportive Care Center, Samsung Medical Center, School of Medicine, Sungkyunkwan University, Seoul 06351, Korea; yohwan.yeo@samsung.com (Y.Y.); jungeunsoph.shin@samsung.com (J.S.); 2 Department of Preventive Medicine, College of Medicine, Seoul National University, Seoul 03080, Korea; shinaesun@snu.ac.kr; 3 Department of Clinical Research Design and Evaluation, Samsung Advanced Institute for Health Science and Technology (SAIHST), Sungkyunkwan University, Seoul 06351, Korea; 4 Department of Digital Health, Samsung Advanced Institute for Health Science and Technology (SAIHST), Sungkyunkwan University, Seoul 06351, Korea; 5 Bucheon Geriatric Medical Center, Bucheon 14478, Gyeonggi-do, Korea; 6 Department of Statistics and Actuarial Science, Soongsil University, Seoul 06978, Korea; hkd@ssu.ac.kr; 7 Department of Medical Statistics, College of Medicine, Catholic University of Korea, Seoul 06951, Korea; ujk8774@catholic.ac.kr; 8 Department of Family Medicine, CHA Gumi Medical Center, Gumi 38295, Gyeongsangbuk-do, Korea; kh1228@chamc.co.kr; 9 Department of International Health Service, Samsung Medical Center, School of Medicine, Sungkyunkwan University, Seoul 06351, Korea; 10 Department of Urology, Uijeongbu Eulji Medical Center, School of Medicine, Eulji University, Uijeongbu-si 11759, Gyeonggi-do, Korea; js.park@eulji.ac.kr

**Keywords:** prostate cancer, prediction, personalized risk, decision aids

## Abstract

Prostate cancer is the fourth most common cause of cancer in men in Korea, and there has been a rapid increase in cases. In the present study, we constructed a risk prediction model for prostate cancer using representative data from Korea. Participants who completed health examinations in 2009, based on the Korean National Health Insurance database, were eligible for the present study. The crude and adjusted risks were explored with backward selection using the Cox proportional hazards model to identify possible risk variables. Risk scores were assigned based on the adjusted hazard ratios, and the standardized points for each risk factor were proportional to the β-coefficient. Model discrimination was assessed using the concordance statistic (c-statistic), and calibration ability was assessed by plotting the mean predicted probability against the mean observed probability of prostate cancer. Among the candidate predictors, age, smoking intensity, body mass index, regular exercise, presence of type 2 diabetes mellitus, and hypertension were included. Our risk prediction model showed good discrimination (c-statistic: 0.826, 95% confidence interval: 0.821–0.832). The relationship between model predictions and actual prostate cancer development showed good correlation in the calibration plot. Our prediction model for individualized prostate cancer risk in Korean men showed good performance. Using easily accessible and modifiable risk factors, this model can help individuals make decisions regarding prostate cancer screening.

## 1. Introduction

Prostate cancer (PC) is the second most common cancer in men worldwide, and overall PC death cases are ranked highest in Asian countries [1]. In Korea, PC has become the fourth most common cancer in men, with a rapid increase in cases in the last decades [2]. In Asian countries, higher grades of PC have been reported compared to the Caucasian population [3,4]. PC screening of serum level of prostate-specific antigen (PSA) [5,6,7] has been used for persons without lower urinary tract symptoms. However, the role of PSA-based screening for PC diagnosis is controversial since it has not led to any significant decrease in PC mortality [8], and the consequent prostate biopsies in patients with elevated PSA level can be harmful. In Korea, early detection of PC by PSA screening has been debated and currently is not included in the national cancer screening program. Professional society recommends shared decision making for PC screening based on individual risk.

Previous models in western countries have adopted screening modalities (PSA or transrectal ultrasonography (TRUS) findings) as predictors to identify accuracy of consequent positive biopsy results [7,9,10,11,12,13]. Similarly, Asian and Korean risk prediction models have been suggested [14,15,16,17]. To indicate the risks to individuals and help them to decide about PC screening, however, population-based predictive tools to evaluate lifestyle risk factors for PC development independent of PSA test or ultrasound are rare. In Korea, a model by Kim et al. consists of lifestyle risk factors for PC incidence based on longitudinal data [15]. However, this model included direct measurement of fasting glucose, which was not easily applicable to individuals estimating PC risk by themselves. Moreover, concerning the changes in health behaviors and PC epidemiology in Korea, a risk prediction model incorporating recent data should be designed to help individuals calculate future risks and make decisions about PC screening.

Therefore, we aimed to develop an individualized risk prediction model for PC using lifestyle and easily accessible variables in clinical settings applicable to all Korean men using representative data from a large population-based cohort study.

## 2. Methods

### 2.1. Database Source

We performed a retrospective cohort study based on the Korean National Health Insurance (KNHI) database (DB), which contains data including inpatient visits, outpatient visits, procedures, and prescription medications covered by the KNHI, a compulsory universal public health insurance system that covers the entire Korean population, except Medicaid beneficiaries in the lowest income bracket (approximately 3% of the population).

The KNHI provides biannual national cardiovascular health check-ups for all beneficiaries aged 20 and older; the KNHI health check-up DB contains medical histories and alcohol, smoking, and exercise habits collected by standardized self-reporting questionnaires. The KNHI also contains a qualification DB (e.g., age, sex, income, region, and type of eligibility), a claims DB (e.g., general information on specifications; statements of consultation; diagnosis statements established by the International Classification of Diseases, 10 revision (ICD-10); and statements of prescriptions), and death information. The KNHI DB has been used widely in various epidemiological and health policy studies [18,19]. Details of the DB profile are described elsewhere [20,21]. This study was approved by the Institutional Review Board of Samsung Medical Center (IRB file no. SMC 2017-12-039, date of approval: 4 January 2018).

### 2.2. Study Population

**Figure 1:**
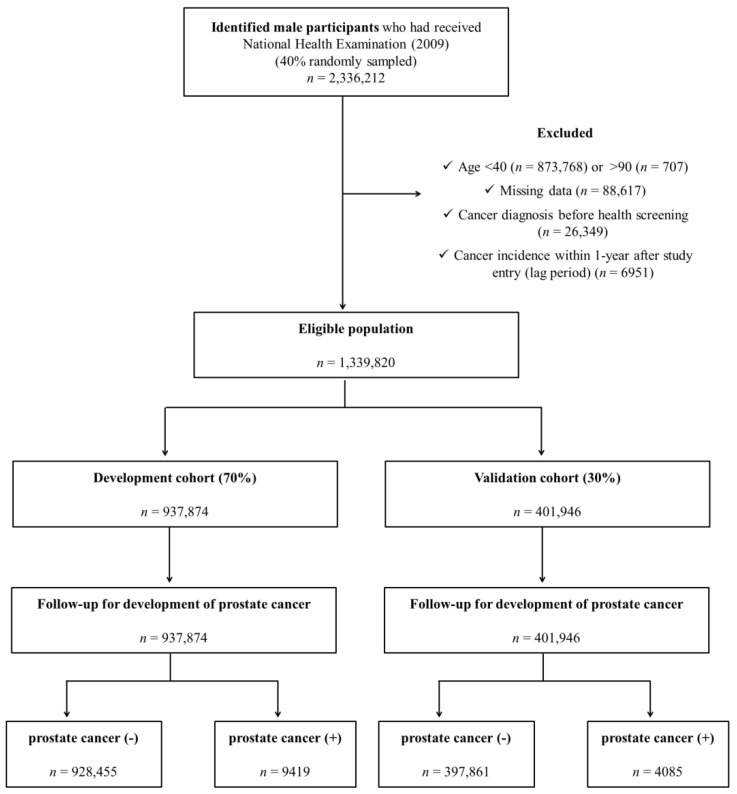
Study design summary.

Among all KNHI beneficiaries, the source population for this study was a random sampling of 40% of the participants who completed health check-ups from 1 January 2009 to 31 December 2009 (accessed on 21 June 2021). Among men who participated in health check-ups in 2009, those younger than 40 years (*n* = 873,768), older than 90 years (*n* = 707) and those with any type of cancer (confirmed with C-code) before health screening (*n* = 26,349) were excluded. In addition, subjects who had been diagnosed with any type of cancer within one year after study enrollment (*n* = 6951) were excluded. Finally, a total of 1,339,820 subjects was eligible (Figure 1).

The original cohort was divided into development and validation datasets. Among 1,339,820 eligible individuals, a total of 937,874, approximately 70% of the total subjects, were selected for the development cohort. For the internal validation cohort, 401,946 subjects, 30% of the total subjects, were extracted from the same DB according to simple random sampling.

### 2.3. Predictor Variables

Among potential risk factors for PC, we selected seven candidates for prediction of PC risk according to a literature review. These were: age; cigarette smoking [22]; alcohol consumption [23,24]; and the metabolic components of body mass index (BMI), regular physical exercise, and presence of DM and hypertension [25,26,27,28].

Demographic information and personal clinical information were obtained, including age, sex, and BMI. The subjects were divided into five groups according to age (40–49, 50–59, 60–69, 70–79, and ≥80 years). We also classified the subjects according to BMI categories recommended by the WHO for Asians (<18.5, 18.5–22.9, 23.0–24.9, 25.0–29.9, and ≥30 kg/m^2^). Smoking intensity was categorized as non-smokers, <10, 10–20, 20–30, and ≥30 pack-years. Alcohol intake was categorized as non-drinkers and light (<15 g/day), moderate (15–30 g/day), and heavy drinkers (>30 g/day). Participants who exercised for one hour on more than three days per week were regarded as performing regular physical exercise.

The presence of comorbidities was defined by diagnostic codes with the prescriptions of relevant medications or by health check-up results: diabetes was defined by ICD-10 codes of E10 through E14 with prescription of at least one antidiabetic medication or with a fasting glucose level of 126 mg/dL or more. The presence of hypertension (ICD-10 codes of I10–I15) was defined by at least one prescription of antihypertensive medication, systolic blood pressure (BP) ≥ 140 mmHg, or diastolic BP ≥ 90 mmHg.

### 2.4. Prostate Cancer as an Outcome

The incidence of PC was defined based on diagnostic codes (i.e., C61) registered after baseline screening with inclusion in a special copayment reduction program for critical illness. In Korea, nearly all people diagnosed with cancer apply for this program since a 5% copayment applies for work-up and treatment for cancer (versus 20–30% for other common diseases). For this reason, cancer incidence in Korea rarely is omitted from this claims DB and is reliable. Among the participants, a total of 13,504 PC cases were enrolled (*n* = 9419 in the development cohort and *n* = 4085 in the validation cohort; Table 1). The claims DB was followed until 31 December 2018, to evaluate the occurrence of PC among the included participants.

### 2.5. Development of Risk Prediction Model

Both crude and age-adjusted risks were explored for possible risk variables, and each variable was included in the model as a categorical variable. A multivariable Cox proportional hazards model was developed employing the times to event between one year after the date of health examination and the date of first PC diagnosis or follow-up termination, whichever came first. The proportional hazards assumption was verified by investigating the Schoenfeld residuals with the logarithm of the cumulative hazards function based on Kaplan–Meier curves. Finally, the best fit risk prediction model was built using backward selection to eliminate non-significant factors among the selected variables.

**Figure 2:**
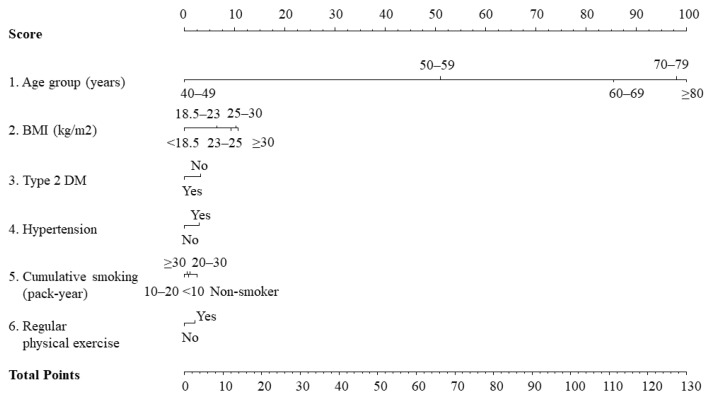
Nomogram for the six-variable prediction model of prostate cancer probability. BMI, body mass index; DM, diabetes mellitus.

After excluding alcohol intake, which was not significant in the model, the six adopted variables (age category, BMI category, cumulative smoking intensity, type 2 DM, hypertension, and regular physical exercise) were applied as weighted risk scores based on the β coefficients for each risk factor in the final Cox proportional hazards model by assigning scores ranging from 0 to 100. Each category of variable according to risk estimation corresponded to a specific point by drawing a line straight up the score axis. The detailed nomogram for PC risk is presented in Figure 2.

### 2.6. Validation of the Risk Prediction Model

Performance of the model was assessed with respect to discrimination and calibration. Model discrimination was evaluated using the concordance statistic (c-statistic) for survival data. ROC curves are corresponding measures with c-statistics explaining the probability of the model to predict the risk of PC for subjects who developed PC compared with those without PC during follow-up. When the discrimination was between 0.60 and 0.80, the prediction model was regarded as good; when the value was higher than 0.80, it was regarded as an excellent model [29]. Internal validation of model discrimination was assessed by calculating the bootstrap optimism-corrected c-statistic with 100 bootstrap replications [30].

Model calibration was assessed by plotting the mean predicted probability against the mean observed probability of PC [31]. Calibration ability refers to the numerical distance between the predicted probabilities to the actual outcomes. The χ^2^ statistic was calculated by first dividing the data into deciles based on predicted probabilities determined by the model. Then, in each decile, the average predicted probabilities were compared with the actual PC risk estimated using the Kaplan–Meier approach. The performance of the developed model was tested on the validation dataset with regard to both discrimination and calibration.

### 2.7. Statistical Analysis

Descriptive data are presented as mean ±SD or frequency and percentage (%). To evaluate the difference between the proportion or means of two variables, Chi-square tests and Student’s *t*-tests were used. Incidence rates of PC were estimated as events per 1000 person-years. A two-sided *p*-value less than 0.05 was considered statistically significant, and all analyses were performed using complete data with SAS (version 9.4; SAS Institute, Cary, NC, USA).

## 3. Results

### 3.1. Clinical Characteristics of the Study Population in the Development and Validation Cohorts

From 1 January to 31 December in 2009, 1,339,820 participants were included in this study. During the mean follow-up period of 8.1 years, 13,504 (1.0%) individuals had a newly identified diagnosis of PC. Compared to participants who did not develop PC during follow-up, the patients who developed PC were older and smoked more (Table 1).

Among the 30% of the study population in the validation cohort (*n* = 401,946), 4085 patients (1.02%) developed PC during the mean follow-up period of 8.1 years (incidence rate: 1.24/1000 person-years). The clinical characteristics of the validation cohort were similar to those of the development cohort including age, BMI, and PC incidence rate (1.36/1000 person-years; Table 1).

### 3.2. Selection of Predictor Variables for the Prediction Model

The crude and adjusted HRs (model 1) for the seven variables of the model are presented in Table 2. The HR was higher based on age group and persisted after adjusting for all listed variables (model 1): sex, regular exercise, BMI, smoking and drinking habits, diabetes, and hypertension. However, alcohol consumption (aHR for heavy drinkers: 0.97, 95% CI: 0.91–1.02) was not a significant factor in Model 1. To determine the best fit model using backward elimination, alcohol consumption was eliminated from the final model (Model 2).

In the final model, a linear trend was observed with normal BMI (18.5–22.9 kg/m^2^) in Asians (at BMI < 18.5 kg/m^2^, aHR: 0.79, 95% CI: 0.68–0.92 and at ≥30 kg/m^2^, aHR: 1.17, 95% CI: 1.02–1.35 (Model 2)). Smokers had a lower risk of PC relative to non-smokers after adjustment for all listed variables (for <10 PY, aHR: 0.94, 95% CI: 0.88–1.01 and for PY ≥ 30, aHR: 0.93, 95% CI: 0.88–0.98). Type 2 DM (aHR: 0.89, 95% CI: 0.84–0.94) and hypertension (aHR: 1.11, 95% CI: 1.07–1.16) were associated with incident PC. Individuals who performed regular physical exercise showed increased risk of PC (aHR: 1.08, 95% CI: 1.03–1.13) after adjusting for all listed variables.

### 3.3. Development of Scores for Prostate Cancer Prediction

**Figure 3:**
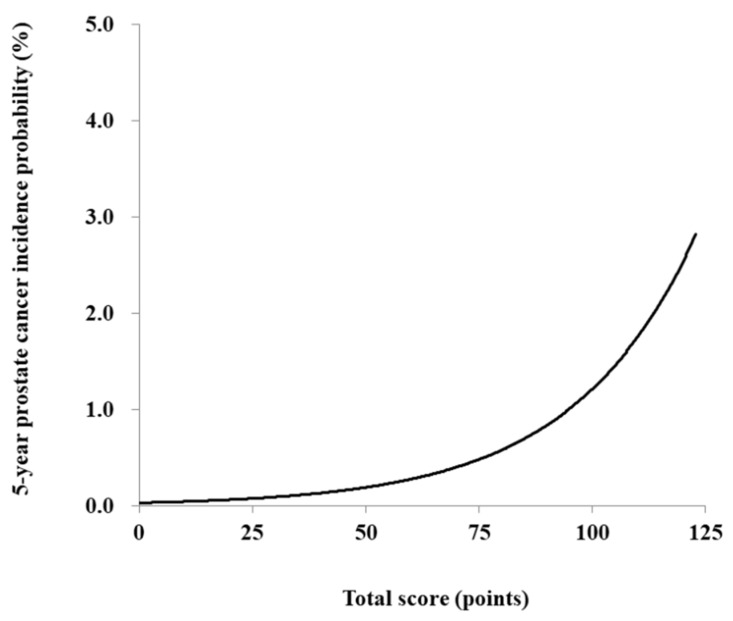
Five-year incidence probability of prostate cancer according to the total score.

**Figure 4:**
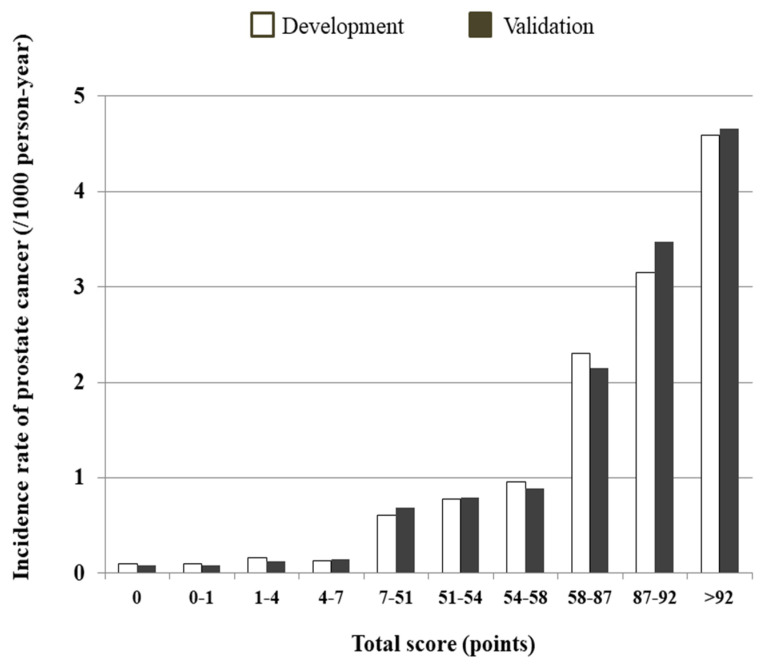
Predicted 5-year prostate cancer incidence rate (per 1000 person-years) by decile score based on the development and validation cohorts.

The risk prediction model for PC was translated into a risk score nomogram (Figure 2). The sums of the scores for the six variables ranged from 0 to 130. Individual risk can be estimated as below; for example, a man aged 60 years (85 points), BMI of 22 kg/m^2^ (6 points), current smoker with more than 20 pack-years (1 point), without type 2 DM (3 points) or hypertension (0 points), and who exercises regularly (2 points) would have 97 points (Table S1). His incidence probability is estimated to be 1.1%. If the total score is greater than 114 points, the incidence probability of PC is >2.0% (Figure 3). The scores showed that the subjects in the highest decile (total score > 92) had the highest incidence rate of 4.595 per 1000 PYs (Figure 4, Table S2).

### 3.4. Validation of the Risk Model

Our risk prediction model showed good discrimination (c-statistic: 0.826, 95% CI: 0.821–0.832). When the performance of the developed model was tested on the validation cohort, the c-statistic for the 5-year prediction of PC incidence was 0.827 (95% CI: 0.819–0.834). The relationship between model prediction and actual PC development correlated well in the calibration plot (Figure S1). Compared with the dashed line representing the performance of an ideal nomogram, the solid line representing the actual outcome was a nearly 45-degree line, indicating that this model corresponded well with actual PC events.

## 4. Discussion

In this study, a risk prediction model for PC in Korea was developed and validated using recent representative data. The performance of our model was good, with competent discrimination demonstrated by a c-statistic of 0.826 (95% CI: 0.821–0.832) and calibration ability. To establish clinically relevant and meaningful models for the general population, the use of easily accessible and modifiable risk factors for PC has been emphasized. Each of the six variables used in the 5-year PC risk model was clinically important and easily applicable.

In Korea, PC incidence has increased rapidly since the 2000s [2]. The increase was interpreted as being due to the increase in life expectancy [32], and partially due to PC screening [33]. Regarding the slight increase in mortality of PC in the 2000s [33], early detection in Korea has been established, but is not included in the national cancer screening program. Nevertheless, PC survival rates have improved significantly since the 2010s, which indicates the possibility of increase in early diagnoses and overall incidence with a consequent increase in prevalent PC cases [34]. PC screening in Korea has been performed widely in private settings, but it is unknown whether men without lower urinary tract symptoms should participate in PC screening. Informed decision making to participate in PC screening has been recommended in Korea.

In contrast to previous models, we explored the risk of PC with clinically available variables prior to PSA measurement or TRUS in clinical settings. To determine the diagnostic ability of PC screening, most previous studies included a screening modality (PSA or TRUS) to predict consequent positive results in prostate biopsy [7,11,12,13,14,15,16,35,36,37,38,39,40,41,42,43,44] (Table S3). Asian and Korean models adopted similar predictors [45]. However, population-based predictive tools evaluating lifestyle risk factors for PC development independent of PSA test or ultrasound should be evaluated to provide individual risk assessments for patients who are concerned about PC screening. Similar to the present study, a previous model in Korea suggested predictors consisting of lifestyle factors applicable to subjects considering PC screening [15]. Regarding the changes in PC epidemiology as well as in health behaviors related to PC, PC risk using recent data representative of Koreans should be identified. Moreover, predictors that require further testing (i.e., laboratory testing of fasting glucose as well as PSA or TRUS) are limited in a clinical setting.

In the present study, the range of risk scores indicating PC probability was relatively narrow; a person with a total score of 122, which was the maximum possible score, had a 5-year PC probability of 2.7%. With the relatively low incidence of PC in Korea compared to western countries, our model demonstrated that persons having total risk scores less than 100 had PC risk less than 1%. For 8-year risk, persons with total risk scores of 100 had increased risk of PC up to 2.7%, which was comparable with the maximum score for 5-year risk (data not shown). Regarding aging, which was the most potent risk factor in our prediction model, further studies should explore when PC screening should be initiated and how often the risk calculation should be used to apply strategies for surveillance of PC.

### 4.1. Predictor Variables for PC Risk

In our model, the independent factors associated with higher PC risk were older age, higher BMI, non-smoking, hypertension, absence of DM, and regular physical exercise. As expected, aging was the most potent factor in our model. Higher BMI was associated with increased risk of PC, which is consistent with a previous Korean prediction model [15] and other epidemiological studies [46,47]. As found in previous studies, persons with hypertension showed a higher risk of PC [28], and DM was inversely associated with PC incidence [26,27].

The results of the associations between lifestyle factors and PC incidence are remarkable. Being a non-smoker and performing regular exercise, which are considered desirable lifestyle factors for cancer prevention, were associated with increased risk of PC in our study. In addition, alcohol consumption was not significantly associated with PC incidence. While this seems perplexing, this is consistent with the results of previous Korean PC prediction models [15]. The previous study also reported decreased risk with smoking and increased risk with moderate to heavy physical activity, and generally no association between alcohol consumption and PC risk (except for decreased risk in heavy drinkers). Previous studies showed mixed results: smoking was associated with decreased risk of PC [22,48], or vice versa [49]; alcohol intake was associated with increased risk of PC [48] or showed no clear association [50,51]; and physical exercise showed an inverse association [15], but was inconsistent [41,52]. One potential explanation for this unexpected association is the effect of PC screening, as those who opt for PC screening are likely to have better health behaviors, i.e., low prevalence of smoking and alcohol consumption and higher prevalence of regular exercise. They also have a likelihood of being diagnosed with early-stage PC relative to non-participants. In addition, since heavy drinkers or smokers are more likely to have other health problems, they could have been excluded unintentionally from our study.

### 4.2. Clinical Implications

Establishment of a risk prediction model to identify individuals with high-risk of PC and recommend screening has become important. Regarding ethnic differences in PC epidemiology, our model including lifestyle variables can serve as a tool to identify an appropriate population for early detection and to maximize the efficacy of screening programs. The Korean Cancer Society and the Korean Foundation for Cancer Research have driven a project to provide aid for self-decisions on participating in PC screening, and our PC risk model for Koreans has been developed. We believe that interactive approaches between healthcare providers and examinees using an easily accessible and visualized risk score can be used for development of health strategies for PC prevention prior to participation in PC screening.

### 4.3. Limitations

The present study had several limitations. First, as we could not link cancer registry data, the outcome ascertainment might be not optimal. The indirect comparison with known PC incidence during the study period showed a slightly higher PC incidence than that reported from the cancer registry data (data not shown). This might be because our population was comprised of screening participants and was more likely to have different health behaviors (i.e., obtaining PSA screening tests) from the general population. Inclusion of screening participants as the study population might be subject to selection bias, as screening participants are likely to have better health status and health behavior. However, medical and health behavior data are only available from the health screening database. In addition, due to free provision of health screening and the high participation rates [53], selection bias likely is not large. Second, the pathologic data or the grading information of incident PC were not available. Recent guidelines have emphasized detecting and treating only clinically significant PC [54,55]. However, when we make inferences between the relatively low PC screening rate and increased presentation of high-grade PC in Korea compared with western countries [3], our model demonstrates future PC risk in Korean men regardless of pathologic aggressiveness.

## 5. Conclusions

We developed a multivariable risk model to predict individual risks of PC incidence in Korean adults. Since making informed decisions for participating in PC screening and avoiding unnecessary biopsies are growing issues, the risk calculated by our model can serve as a tool for discussion between healthcare providers and examinees in clinical setting. Further studies to identify high-risk subjects who could benefit from PC screening and to maximize the efficacy of PC screening are necessary. Health strategies to reduce future PC risk according to individual scores estimated by lifestyle factors should be followed.

## Figures and Tables

**Table 1:** Baseline characteristics according to prostate cancer incidence in the development and validation cohorts.

	Development Cohort (*n* = 937,874)				Validation Cohort (*n* = 401,946)			
	Total (*n* = 937,874)	Prostate Cancer Did Not Develop (*n* = 928,455)	Prostate Cancer Developed ‡ (*n* = 9419)	*p*-Value †	Total (*n* = 401,946)	Prostate Cancer Did Not Develop (*n* = 397,861)	Prostate Cancer Developed ‡ (*n* = 4085)	*p*-Value †
Age (years) (*n*, %)				<0.001				<0.001
40–49	388,121 (41.4)	387,716 (41.8)	405 (4.3)		165,351 (41.1)	165,205 (41.5)	146 (3.6)	
50–59	287,665 (30.7)	285,695 (30.8)	1970 (20.9)		123,552 (30.7)	122,717 (30.8)	835 (20.4)	
60–69	176,198 (18.8)	172,001 (18.5)	4197 (44.6)		75,992 (18.9)	74,132 (18.6)	1860 (45.5)	
70–79	75,854 (8.1)	73,284 (7.9)	2570 (27.3)		32,750 (8.2)	31,624 (8.0)	1126 (27.6)	
≥80	10,036 (1.1)	9759 (1.1)	277 (2.9)		4301 (1.1)	4183 (1.1)	118 (2.9)	
BMI (kg/m^2^) (*n*, %)				0.015				0.161
<18.5	18,941 (2.0)	18,762 (2.0)	179 (1.9)		8026 (2.0)	7951 (2.0)	75 (1.8)	
18.5–23	299,791 (32.0)	296,811 (32.0)	2980 (31.6)		128,095 (31.9)	126,827 (31.9)	1268 (31.0)	
23–25	264,713 (28.2)	261,969 (28.2)	2744 (29.1)		113,960 (28.4)	112,734 (28.3)	1226 (30.0)	
25–30	328,322 (35.0)	325,021 (35.0)	3301 (35.1)		140,630 (35.0)	139,218 (35.0)	1412 (34.6)	
≥30	26,107 (2.8)	25,892 (2.8)	215 (2.3)		11,235 (2.8)	11,131 (2.8)	104 (2.6)	
Diabetes mellitus (*n*, %)				<0.001				<0.001
No	808,934 (86.3)	801,107 (86.3)	7827 (83.1)		346,117 (86.1)	342,725 (86.1)	3392 (83.0)	
Yes	128,940 (13.8)	127,348 (13.7)	1592 (16.9)		55,829 (13.9)	55,136 (13.9)	693 (17.0)	
Hypertension (*n*, %)				<0.001				<0.001
No	594,415 (63.4)	590,060 (63.6)	4355 (46.2)		253,876 (63.2)	251,997 (63.3)	1879 (46.0)	
Yes	343,459 (36.6)	338,395 (36.5)	5064 (53.8)		14,8070 (36.8)	145,864 (36.7)	2206 (54.0)	
Smoking (pack-years) (*n*, %)				<0.001				<0.001
Non-smoker	299,939 (32.0)	296,040 (31.9)	3899 (41.4)		128,582 (32.0)	126,871 (31.9)	1711 (41.9)	
<10	135,422 (14.4)	134,437 (14.5)	985 (10.5)		58,177 (14.5)	57,749 (14.5)	428 (10.5)	
10–20	179,181 (19.1)	177,950 (19.2)	1231 (13.1)		76,926 (19.1)	76,378 (19.2)	548 (13.4)	
20–30	156,183 (16.7)	155,011 (16.7)	1172 (12.4)		66,644 (16.6)	66,168 (16.6)	476 (11.6)	
≥30	167,149 (17.8)	165,017 (17.8)	2132 (22.6)		71,617 (17.8)	70,695 (17.8)	922 (22.6)	
Alcohol drinking (*n*, %)				<0.001				<0.001
Non-drinker	334,025 (35.6)	329,745 (35.5)	4280 (45.4)		143,868 (35.8)	142,000 (35.7)	1868 (45.7)	
Light drinker	305,135 (32.5)	302,297 (32.6)	2838 (30.1)		130,113 (32.4)	128,910 (32.4)	1203 (29.4)	
Moderate drinker	167,680 (17.9)	166,429 (17.9)	1251 (13.3)		71,824 (17.9)	71,237 (17.9)	587 (14.4)	
Heavy drinker	131,034 (14.0)	129,984 (14.0)	1050 (11.2)		56,141 (14.0)	55,714 (14.0)	427 (10.5)	
Regular exercise (*n*, %)				<0.001				<0.001
No	726,902 (77.5)	720,022 (77.6)	6880 (73.0)		311,595 (77.5)	308,614 (77.6)	2981 (73.0)	
Yes	210,972 (22.5)	208,433 (22.5)	2539 (27.0)		90,351 (22.5)	89,247 (22.4)	1104 (27.0)	

BMI, body mass index, ‡ Prostate cancer developed within 8.1 years of mean follow-up, † Tested using chi-square test for categorical variables.

**Table 2:** Hazard ratio and 95% CI for prostate cancer incidence.

	Number of Subjects	Events	Follow-Up (Person-Years)	IR	Crude HR (95% CI)	*p*-Value	Model 1 aHR (95% CI)	*p*-Value	Model 2 aHR (95% CI)	*p*-Value
Age (years)						<0.001		<0.001		<0.001
40–49	388,121	405	3,210,912.44	0.13	1.00 (ref.)		1.00 (ref.)		1.00 (ref.)	
50–59	287,665	1970	2,359,084.73	0.84	6.62 (5.95, 7.37)		6.52 (5.85, 7.26)		6.53 (5.87, 7.27)	
60–69	176,198	4197	1,401,358.77	3.00	23.84 (21.52, 26.39)		23.05 (20.77, 25.58)		23.18 (20.89, 25.71)	
70–79	75,854	2570	551,444.31	4.66	37.66 (33.92, 41.83)		36.43 (32.70, 40.59)		36.70 (32.95, 40.86)	
≥80	10,036	277	58,566.1	4.73	40.07 (34.39, 46.69)		39.19 (33.53, 45.82)		39.57 (33.87, 46.23)	
BMI (kg/m^2^)						0.024		<0.001		<0.001
<18.5	18,941	179	140,767.92	1.27	1.04 (0.89, 1.21)		0.79 (0.68, 0.92)		0.79 (0.68, 0.92)	
18.5–23	299,791	2980	2,401,168.63	1.24	1.00 (ref.)		1.00 (ref.)		1.00 (ref.)	
23–25	264,713	2744	2,150,648.91	1.28	1.03 (0.97, 1.08)		1.11 (1.06, 1.17)		1.11 (1.06, 1.17)	
25–30	328,322	3301	2,676,288.76	1.23	0.99 (0.94, 1.04)		1.16 (1.10, 1.22)		1.16 (1.10, 1.22)	
≥30	26,107	215	212,492.13	1.01	0.82 (0.71, 0.94)		1.17 (1.02, 1.35)		1.17 (1.02, 1.35)	
Diabetes mellitus (yes)						<0.001		<0.001		<0.001
No	808,934	7827	6,569,175.73	1.19	1.00 (ref.)		1.00 (ref.)		1.00 (ref.)	
Yes	128,940	1592	1,012,190.61	1.57	1.33 (1.26, 1.40)		0.89 (0.84, 0.94)		0.89 (0.84–0.94)	
Hypertension (yes)						<0.001		<0.001		<0.001
No	594,415	4355	4,855,107.99	0.90	1.00 (ref.)		1.00 (ref.)		1.00 (ref.)	
Yes	343,459	5064	2,726,258.35	1.86	2.08 (2.00, 2.16)		1.12 (1.07, 1.17)		1.11 (1.07, 1.16)	
Smoking (pack-years)						<0.001		0.037		0.015
Non-smoker	299,939	3899	2,419,348.09	1.61	1.00 (ref.)		1.00 (ref.)		1.00 (ref.)	
<10	135,422	985	1,106,211.8	0.89	0.55 (0.52, 0.59)		0.94 (0.88, 1.01)		0.94 (0.88,1.01)	
10–20	179,181	1231	1,460,326.63	0.84	0.52 (0.49, 0.56)		0.92 (0.86, 0.98)		0.91 (0.86, 0.97)	
20–30	156,183	1172	1,267,185.13	0.92	0.58 (0.54, 0.61)		0.95 (0.89, 1.02)		0.95 (0.89, 1.01)	
≥30	167,149	2132	1,328,294.69	1.61	1.00 (0.95, 1.05)		0.94 (0.89, 0.99)		0.93 (0.88, 0.98)	
Alcohol drinking						<0.001		0.146		
Non-drinker	334,025	4280	2,667,697.39	1.60	1.00 (ref.)		1.00 (ref.)			
Light drinker	305,135	2838	2,487,174.59	1.14	0.71 (0.68, 0.75)		1.01 (0.96, 1.06)			
Moderate drinker	167,680	1251	1,366,608.97	0.92	0.57 (0.54, 0.61)		0.94 (0.88, 1.01)			
Heavy drinker	131,034	1050	1,059,885.39	0.99	0.62 (0.58, 0.66)		0.93 (0.87, 1.00)			
Regular exercise (yes)						<0.001		0.001		0.001
No	726,902	6880	5,868,942.24	1.17	1.00 (ref.)		1.00 (ref.)		1.00 (ref.)	
Yes	210,972	2539	1,712,424.1	1.48	1.26 (1.21, 1.32)		1.08 (1.03, 1.13)		1.08 (1.03, 1.13)	

Abbreviations: IR, incidence rate per 1000 person-years; HR, hazard ratio; CI, confidence interval; aHR, adjusted hazard ratio; BMI, body mass index;. Model 1: adjusted for all possible predictor variables listed in the table. Model 2: adjusted for predictor variables selected by backward selection.

## Data Availability

The datasets and statistical analysis used for the current study can be repeated only by re-analysis at the data center on reasonable request.

## Supplementary Materials

The following are available online at https://www.mdpi.com/article/10.3390/jpm12010002/s1, Table S1: Scores for each risk factor category, Table S2: Predicted incidence rate (per 1000 person-years) based on the development and validation cohorts, Table S3: Summary of previous risk prediction models for prostate cancer incidence, Figure S1: Calibration plots between predicted and observed 5-year prostate cancer development.
