# Supplementary material for: Personalized 5-Year Prostate Cancer Risk Prediction Model in Korea Based on Nationwide Representative Data
Source: J Pers Med. 2021 Dec 21;12(1):2. doi: 10.3390/jpm12010002 (PMC8780119; doi:10.3390/jpm12010002)
Supplement: Supplementary file 1 [file jpm-12-00002-s001.zip › jpm-1470382-supplementary.pdf]

Journal of *Personalized Medicine*

Supplementary Materials

Table S1. Scores for each risk factor category.

No.	Variables	Categories and scores				
1	Age (years)	40–49	50–59	60–69	70–79	≥80
		0	51	85	98	100
2	Smoking (pack-year)	Non-smoker	<10	10–20	20–30	≥30
		3	1	0	1	0
3	BMI (kg/m²)	≥30	25–30	23–25	18.5–23	<18.5
		11	10	9	6	0
4	DM	Yes	No			
		0	3			
5	Hypertension	No	Yes			
		0	3			
6	Regular exercise	No	Yes			
		0	2			

BMI, body mass index; DM, diabetes mellitus.

Table S2. Predicted incidence rate (per 1000 person-years) based on the development and validation cohorts.

Decile		1	2	3	4	5	6	7	8	9	10
Points		0	0–1	1–4	4–7	7–51	51–54	54–58	58–87	87–92	92
Incidence rate of prostate cancer (per 1000 person-years)	Development cohort	0.104	0.099	0.165	0.130	0.611	0.781	0.962	2.305	3.155	4.595
	Validation cohort	0.080	0.086	0.120	0.145	0.690	0.792	0.892	2.145	3.475	4.659

Table S3. Summary of previous risk prediction models for prostate cancer incidence.

Author	Year at enrollment	Country	Study population	Follow-up	Predictor variables	Primary outcome	Model performance
1. Ankerst et al., 2014 [36]	2006–2012	PCPTRC (Prostate Cancer Prevention Trial risk calculator)	Total *n* = 6664 PC cases = 1196	NA	Age, race PSA, % free PSA level DRE findings Previous prostate biopsy Family history of PC	PC/High-grade PC	AUC = 0.568 AUC = 0.744 (high-grade PC)
2. Ankerst et al., 2018 [12]	2006–2017	North Americans PBCG (Prostate Biopsy Collaborative Group)	Total *n* = 5992 PC cases = 3009	NA	Age, race PSA level DRE results Previous negative biopsy Family history of PC	PC	AUC = 0.755
3. Chen et al., 2016 [37]	2011–2013	China	Total *n* = 924 PC cases = 338	NA	Age PSA, free PSA ratio Prostate volume, DRE findings	PC/High-grade PC	AUC = 0.801 AUC = 0.826 (high-grade PC)
4. Chun et al., 2007 [38]		Germany	Total *n* = 1162 PC cases = 485	NA	Age PSA, % free PSA level Prostate volume	PC	AUC = 0.770
5. Finne et al., 2004 [39]	1991–1999	ERSPC (European Randomized Study of Screening for Prostate Cancer)	Total *n* =1775 PC cases = 402	NA	Age PSA, % free PSA level DRE findings	PC	AUC = 0.764
6. Grönberg et al., 2015 [40]	2012–2013	Sweden STHLM3 (Stockholm 3 study)	Total *n* = 32,823 PC cases = 429	NA	Age Combined biomarker score (PSA, fPSA, iPSA,hK2,MSMB,MIC1,genetic markers), DRE findings Prostate volume Previous prostate biopsy Family history of PC	PC	AUC = 0.740
7. Karakiewicz, et al., 2005 [35]	1990–1998	Canada	Total *n* = 4193 PC cases = 1477	NA	Age PSA, % free PSA level DRE findings	PC	AUC = 0.770
8.Kim et al., 2018 [15]	1996–1997	Korea	Total *n* = 1,179,172 PC cases = 2747	8 years	Age Height, BMI Fasting glucose level Family history of cancer Meat consumption (frequency per week) Alcohol intake (g/day) Smoking status Physical activity	PC incidence	AUC = 0.887
9. Nam et al., 2018 [11]	2009–2014	US	PC cases = 5638 High-grad PC cases = 629	NA	Age, race PSA, free/total PSA ratio, DRE findings Family history of PC AUA symptom scores	High-grade PC	AUC = 0.740
10. Park et al., 2011 [14]	2004–2008	Korea (KPCRC, Korean prostate cancer risk calculator)	Total *n* = 602 PC cases = 172	NA	Age DRE findings PSA level Prostate transitional zone volume	PC	AUC = 0.910
11. Roffman et al., 2018 [41]	1997–2015	US	Total *n* = 71,194 PC cases = 1171	4 years (cut-off)	Age, race BMI Smoking status Vigorous exercise Diabetes, hypertension, Emphysema, Asthma Stroke	PC incidence	AUC = 0.730

					Heart disease score (angina, heart attack, other heart complications)		
12. Roobol et al., 2012 [42]	1991–1999	Netherland ERSPC (European Randomized Study of Screening for PC), Rotterdam section	Total n = 3624 PC cases = 885	NA	PSA level DRE findings TRUS results, Prostate volume	PC/High-grade PC	AUC = 0.860 AUC = 0.790 (high-grade PC)
13. Suzuki et al., 2006 [16]	2000–2003	Japan	Total n= 834 PC cases = 241	NA	Age PSA level, free/total PSA ratio Prostate volume DRE findings	PC	AUC = 0.818
14.Thompson et al., 2006 [13]	1993–2003	Placebo arm of PCPT (Prostate Cancer Prevention Trial)	n = 5519 PC cases = 1211	NA	Age, Race PSA level DRE findings Previous prostate biopsy Family history of PC	PC/High-grade PC	AUC = 0.702 AUC = 0.698 (high-grade PC)
15. Wu et al., 2016 [43]	2006–2010	China	Total n = 1059 PC cases = 480	NA	Age PSA, % free PSA level TRUS results, prostate volume DRE findings	PC/High-grade PC	AUC = 0.901 AUC = 0.814 (high-grade PC)
16. Wu et al., 2019 [44]	2012–2013	China	Total n= 635 PC cases = 272	NA	Age PSA, % free PSA level Prostate volume Prostate Health Index (phi)	PC/High-grade PC	AUC = 0.895 AUC = 0.890 (high-grade PC)

PSA, prostate specific antigen; fPSA, free prostate specific antigen; iPSA, intact prostate specific antigen; hk2, human kallikrein-2; MSMB, microseminoprotein-beta; MIC1, macrophage inhibitory cytokine-1; AUC, area under curve; PC, prostate cancer; DRE, digital rectal exam; TRUS, trans-rectal ultrasonography; BMI, body mass index; NA, not applicable.

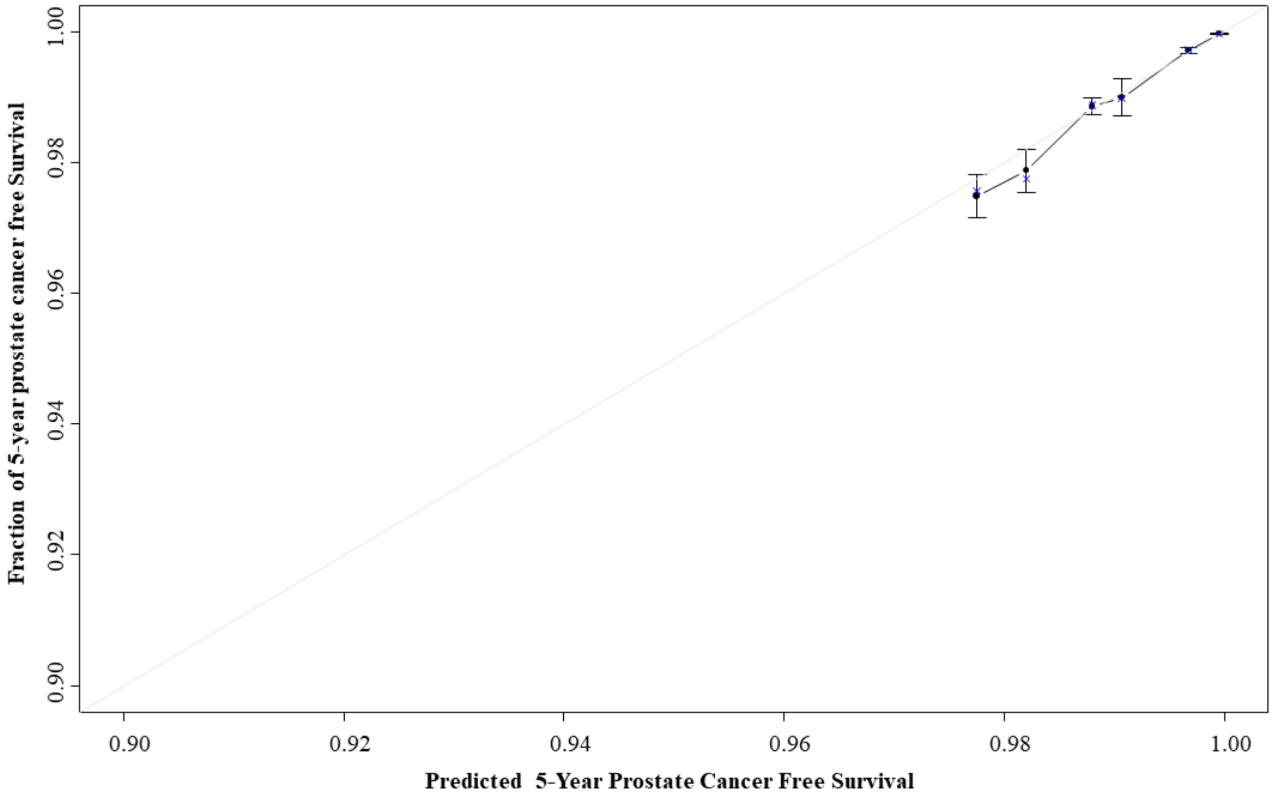

Figure S1. Calibration plots between predicted and observed 5-year prostate cancer development.
